# Intravenous immunoglobulins may prevent prednisone-exacerbation in myasthenia gravis

**DOI:** 10.1038/s41598-020-70539-4

**Published:** 2020-08-11

**Authors:** Laura Díez-Porras, Christian Homedes, Maria Antonia Alberti, Valentina Vélez-Santamaría, Carlos Casasnovas

**Affiliations:** 1 Neuromuscular Unit. Department of Neurology, Bellvitge University Hospital, Feixa Llarga Street s/n, L’Hospitalet del Llobregat, 08907 Barcelona, Spain; 2 Neurometabolic Diseases Group, Bellvitge Biomedical Research Institute (IDIBELL), 199 Granvia de l’Hospitalet, L’Hospitalet de Llobregat, 08908 Barcelona, Spain; 3 Center for Biomedical Research on Rare Diseases (CIBERER), ISCIII, 3-5 Monforte de Lemos, Pabellón 121, 28029 Madrid, Spain

**Keywords:** Neuromuscular disease, Neuroimmunology

## Abstract

Corticosteroids may produce a paradoxical worsening of myasthenia gravis (MG) symptoms within the first weeks of treatment. We therefore wanted to assess the hypothesis that a prior infusion of intravenous immunoglobulin (IVIG) may have a protective effect. Our primary objectives were to show that the coadministration of immunoglobulins and glucocorticoids is safe and effective for controlling myasthenic symptoms, and to compare the exacerbation rate with this approach and historical practice without IVIG. We recruited 45 patients with generalized MG who required corticosteroids for the first time and we gave all IVIG before starting the full doses of prednisone. Monitoring was performed with validated scales, questionnaires, and blood tests over a 6-week period. Only 4.4% had severe adverse effects related to IVIG and 86.7% improved clinically. Notably, only 2.2% had a paradoxical symptom exacerbation in the first weeks of starting prednisone, which was statistically lower than the 42% reported in a historical series. We conclude that adjuvant therapy with IVIG when starting prednisone for the first time in patients with generalized MG is safe and effective. Given that the rate of paradoxical worsening was lower than that previously reported, the addition of IVIG may have a protective effect against such exacerbations.

## Introduction

Myasthenia gravis (MG) is a rare disease that affects 15–32.89 people per 100,000^1–4^. Patients with MG classically present with characteristic symptoms of muscle weakness and fatigability, particularly of the ocular, facial, oropharyngeal, limb, and respiratory muscles. The disease is mediated by circulating organ-specific antibodies against skeletal muscle receptors and neuromuscular junction proteins that alter the neuromuscular transmission^2, 3^.

The first immunosuppressive agents used in the treatment of MG were corticosteroids^5–14^. Prednisone is generally added to anticholinesterase therapy when these alone do not control symptoms. Although there is a lack of controlled prospective randomized clinical trials demonstrating its efficacy, several clinical studies have shown that daily administration of high-dose corticosteroids can significantly improve symptoms^5, 6, 10, 12, 15–18^. However, paradoxical exacerbation of MG symptoms by prednisone is a well-described phenomenon, especially in the first weeks after starting treatment^7, 19, 20^. There is a wide spectrum of severity with prednisone-induced exacerbations, with reports ranging from mild cases to deterioration resulting in death due to respiratory failure^9, 20^. Several hypotheses have been proposed to explain the mechanism of steroid-induced exacerbation. For example, antibodies released by degraded lymphocytes may increase cholinesterase activity at the neuromuscular junction and increase the immune response^9, 18, 20, 21^. However, the underlying primary mechanism is not yet clearly established and the optimum treatment to achieve maximum effect with minimum side effects is still under debate. Some authors have stated that prednisone-induced exacerbations can be avoided by using a regime that rapidly escalates to a high dose rather than starting immediately at the target dose^6, 14, 18^, but this approach also lacks scientific evidence.

Other treatments that have demonstrated efficacy in MG are plasma exchange (PLEX) and intravenous immunoglobulins (IVIG)^22^. PLEX is the comparatively more invasive procedure of the two, requiring trained personnel and specific equipment that is not routinely available in all hospitals. IVIG, which was first used for MG in 1984 by Gajdos et al.^23^, has been proven to be effective in several studies. In patients who deteriorated from moderate to severe MG, IVIG was superior to placebo at 14 days^24^. In two trials, IVIG was also shown to be as effective as PLEX in controlling MG exacerbations after 14–15 days of treatment^25, 26^. Another clinical trial showed that a 2 g/kg dose of IVIG was not superior to a 1 g/kg dose when treating exacerbations^27^. A retrospective study of myasthenic crises determined that patients treated with PLEX had a better ventilatory status and functional outcome than those treated with IVIG^28^. Another 2016 article in a pediatric population reported that combining IVIG with prednisone controlled symptoms faster and shortened hospital stays^29^. The European Federation of the Neurological Societies recommends using IVIG to treat exacerbations or for maintenance in some cases of refractory MG^30^. Currently there is class A evidence to use IVIG to treat exacerbations of MG^31^. In addition, some authors recommend IVIG or PLEX before thymectomy to prevent a myasthenic crisis^22^, although a recent clinical trial did not demonstrate such benefit for IVIG^32^. Other authors suggest that treatment with IVIG or PLEX can prepare patients who are to receive immunosuppressives^22, 33^, and an international consensus on the management of MG has stated that treatment with IVIG and PLEX before starting corticosteroids is appropriate in an effort to prevent or minimize exacerbations^34^. However, there is currently a lack of data to justify the use of IVIG before starting immunosuppressive treatment, and as such, its use remains “off-label” (evidence level = 5)^31^.

In our opinion, IVIG infusions can improve the safety of starting prednisone at maximum doses for the first time by reducing the rate of paradoxical steroid-induced exacerbations. Therefore, we aimed to demonstrate whether combination treatment with IVIG and prednisone is safe and effective in patients with generalized MG receiving remission doses of corticosteroids for the first time. This treatment approach was compared with standard treatment in a historical cohort to assess differences in the proportion of patients developing paradoxical exacerbation in the first 6 weeks. This has previously been reported by Bae et al. to be 42%.

## Subjects and methods

This was a single centre, prospective, monitoring, post-authorization study. In accordance with the Declaration of Helsinki, the study was conducted following a protocol approved by the institutional review board of the Clinical Research and Clinical Trials Unit (UCICEC) of Bellvitge Biomedical Research Institute (IDIBELL). All participants signed an informed written consent form, and investigators maintained participant anonymity by using codes that were stored in a locked area. UCICEC IDIBELL monitored the study regularly. Annual reports were sent to two local drug regulatory agencies: the AEMPS (Spanish Agency for Medicines and Health Products; code IDI-INM-2016-01) and the Department of Health of the Autonomous Government of Catalonia (CCP-INM-2016-01).

We recruited all consecutive patients with generalized MG of grades IIa to V according to the Myasthenia Gravis Foundation of America (MGFA) clinical classification system^35^ that attended our hospital between April 2016 and January 2019. Only those meeting the eligibility criteria were included (the inclusion and exclusion criteria are specified in Supplementary Table S1). As other international recent studies^32, 36^, we also included mild generalized MG patients (MGFA class IIA of the MGFA functional class and minimal manifestations category of the MGFA Post-Intervention Status at study inclusion).

**Figure 1:**
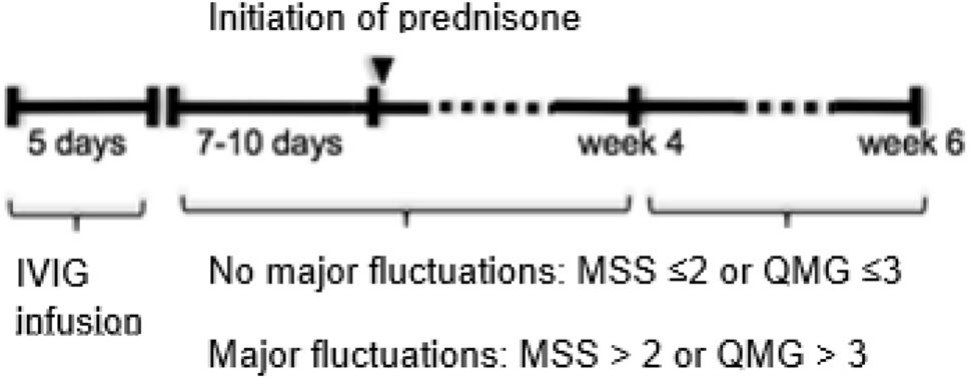
Summary figure of the study with MSS and QMG criteria of significant fluctuations. MSS scale, used by Bae et al. and that we have used to measure significant fluctuations as a primary objective.

Patients received one round of IVIG (0.4 g/kg/day for 5 days) and after 7–10 days they were started on full therapeutic doses of prednisone (1 mg/kg/day or 0.75 mg/kg/day in patients with comorbidities). The 7–10 day wait before starting prednisone was to the exclusion of any patients with highly fluctuating MG who could be falsely interpreted as having prednisone exacerbations if recruited (Fig. 1). The treatment received by patients was in line with the clinical guidelines for MG^30, 34, 37^.

We adopted a policy of close follow-up with a battery of internationally validated scales for MG and with standardized quality of life scales and questionnaires^38–44^ (see Supplementary appendix). The study visits took place before or during IVIG infusion but before starting prednisone (visit 1), as well as at 4 weeks (visit 5) and 6 weeks (visit 7) after starting prednisone. In addition, telephone consultations were performed at 1, 2, 3, and 5 weeks after starting prednisone (visits 2, 3, 4, and 6) to detect minor paradoxical deteriorations or any adverse effects. If patients noticed any deterioration or were concerned, extra visits were made available. We also performed blood tests and autoimmune testing, which included anti-acetylcholine receptor antibody and anti-striated muscle antibody titres at visits 1, 5, and 7 anti-MuSK antibody titres at visit 1. These blood tests were performed at the laboratory of Bellvitge University Hospital (HUB).

At visits 5 and 7, we assessed whether the patient was better, worse, or the same as at visit 1 based on the scores from the evaluation scales. Deterioration was defined as a decrease of > 2 points in the Myasthenia Gravis Severity Scale (MSS)^19, 28^ (Supplementary Table 2) or an increase > 3 points in the Quantitative Myasthenia Gravis (QMG)^38^. Improvement in MG was defined as an increase of more than 2 points in the MSS score or a decrease greater than 3 points in the QMG score. The patient was categorized as stable if the difference in scores was ≤ 2 or ≤ 3 on the MSS and QMG scales, respectively (Fig. 1).

All statistical analyses were performed using IBM SPSS version 23.0 (IBM Corp., Armonk, NY, USA). The results are presented as numbers and percentages. The Fisher exact test was used to compare categorical variables between our series and the historical series published by Bae et al. to assess differences in the development of glucocorticoid-induced exacerbations. Statistical significance was set at p < 0.05.

## Results

**Figure 2:**
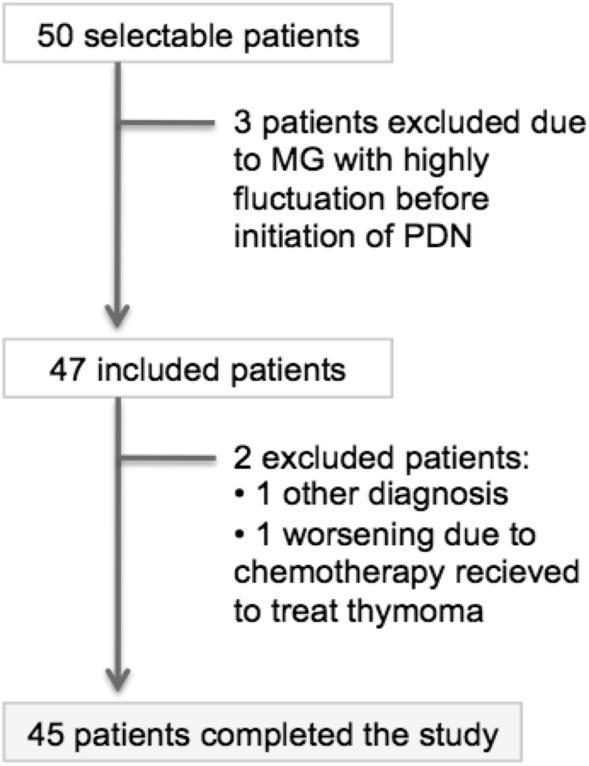
Diagram showing the patients included in the study.

We recruited 47 patients in total. Two patients left the study: one who was misdiagnosed of MG but had progressive bulbar paralysis and one who had worsening of MG secondary to adverse effects to chemotherapy started for treatment of thymoma (Fig. 2).

Of the 45 patients included in the analysis, most were men and had an onset of MG after 50 years of age. None of the patients had anti-MuSK antibodies and only four were double seronegative. More than half of the patients had mild MG (MGFA class IIa or IIb at clinical onset and MM-1 and MM-2 of the MGFA at study inclusion) and only two patients presented with MGFA class V. One third of the sample had undergone thymectomy, and in two patients, this was because of a thymoma (Table 1).

**Table 1:** Demographic and clinical features.

	Total 45
**Sex**	
Man, n (%)	30 (66.67)
Woman, n (%)	15 (33.33)
**Age (years)**	
Median	69
Minimum	26
Maximum	85
**Mean onset age (years)**	65.22 ± 16.31
**Age at onset (%)**	
≥ 50 years	40 (88.9)
< 50 years	6 (13.3)
**Onset symptom (%)**	
Ocular	34 (75.6)
Bulbar	9 (20)
Spinal	1 (2.2)
Respiratory	1 (2.2)
**Pre-treatment MGFA class**	
IIA, n (%)	10 (22.2)
IIB, n (%)	14 (31.1)
IIIA, n (%)	5 (11.1)
IIIB, n (%)	9 (20)
IV, n (%)	5 (11.1)
V, n (%)	2 (4.4)
**Pre-treatment MGFA-PIS category**	
Minimal manifestations 0 (MM-0)	3 (6.7)
Minimal manifestations-2 (MM-2)	2 (4.4)
Minimal manifestations 3 (MM-3)	7 (15.6)
Exacerbation (E)	33 (73.3)
**Pre-treatment MSS (mean ± standard deviation)**	12.64 ± 1.64
**Thymectomized, n (%)**	15 (33.3)
**Thymus, n (%)**	
Thymic hyperplasia	4 (8.9)
Atrophy or thymic remains	9 (20)
Thymoma	2 (4.4)
Chest tomography not suggestive of thymoma	30 (66.7)
**Antibodies**	
AntiRAch, n (%)	41 (91.1)
AntiMusk, n (%)	0 (0)
Double seronegatives	4 (8.9)
Anti striated muscle, n (%)	22 (48.9)

Regarding the adverse effects of IVIG, more than half of the patients had mild side effects, with mild asymptomatic transaminitis being most common (aspartate transaminase and/or alanine aminotransferase values up to 1.2 μkat/L). Only two patients had severe adverse effects: one had a deep venous thrombosis that required anticoagulation and one developed a subclinical pulmonary thromboembolism that also required anticoagulation (Table 2). Both of these patients had received the IVIg during hospitalization for myasthenic crisis. No patient with mild generalized MG treated with IVIG had severe adverse effects. More than half of the patients had side effects attributable to prednisone, with the most frequent being insomnia, irritability, hyperglycaemia, and uncontrolled hypertension (Table 2).

**Table 2:** Advers effects (AE) to intravenous immunoglobulins (IVIg) and prednisone.

AE to IVIG	Count (percentage %)	Total	AE to prednisone	Count (percentage %)	Total
No	17 (37.8)	17 (37.8)	No	20 (44.4)	20 (44.4)
Transaminitis	21 (46.7)	28 (62.2)	Insomnia	8 (17.8)	25 (55.6)
Headache	18 (40)		Irritability/mood change	6 (13.3)	
Flu-like	2 (4.4)		High blood pressure	4 (8.9)	
Deep vein thrombosis	1 (2.2)		Hyperglycemia	4 (8.9)	
Varicose phlebitis	1 (2.2)		Weight gain	3 (6.7)	
Stomack flu	1 (2.2)		Edemas	1 (2.2)	
Pulmonary embolism	1 (2.2)		Other	4 (8.9)	
Cutaneous rash	1 (2.2)				
Dizziness	1 (2.2)				

Concerning the response to combined therapy with IVIG and prednisone, 39 patients (86.7%) of the sample had a clear clinical improvement at week 4 and only one patient (2.2%) had exacerbation of MG symptoms in the first weeks of prednisone treatment based on Bae et al.’s criteria with the MSS. Using more sensitive criteria to detect significant fluctuations in MG (QMG, MG-Composite scale), we identified two additional patients with significant deterioration.

These three cases of paradoxical exacerbation required a second round of IVIG to achieve clinical improvement or stabilization, and they were subsequently maintained on high doses of prednisone. Their conditions also improved at subsequent visits. Only one patient did not respond to treatment and remained stable despite the prednisone dose and a round of IVIG. Therefore, when analyzing the efficacy of combined therapy, 91.1% of patients responded to treatment at 6 weeks (Table 3).

**Table 3:** Comparison between the steroid exacerbated and non-exacerbated myasthenia gravis groups between our series and Bae et al. series.

	Our series		Bae et al.	
	Exacerbated (n = 1)	Non-exacerbated (n = 44)	Exacerbated (n = 23)	Non-exacerbated (n = 32)
**Demographic features**				
Age (years)	56	66.43 ± 16.38	52.3 ± 13.4	41.1 ± 15.4
Male/female ratio	1	29/15	8/15	12/20
Age at onset (years)	56	65.22 ± 16.32	48.5 ± 14.0	39.8 ± 15.4
**Pre-treatment clinical status of MG**				
Pre-treatment dose of piridostigmine (mg)	240	152.14 ± 94.96	259.8 ± 131.9	215.6 ± 115.1
MSS score	12	12.66 ± 1.66	9.4 ± 2.0	12.5 ± 1.6
Functional MG scale score	4	3.22 ± 0.82	4.4 ± 0.6	3.4 ± 0.6
Thymomatous MG (n)	0	2	8	14
Thymectomized MG (n)	0	15	13	17
**Laboratory findings**				
Patients with AChR-Ab (%)	1	90.9%	–	–
**Outcome after use of PDN**				
Reason for PDN use, bulbar symtoms (n)	1	29	23	18
Total PDN dose	100	62.5 ± 15.87	66.7 ± 11.6	62.3 ± 14.5
Dose per body weight (mg/kg)	1	0.9 ± 0.15	1.3 ± 0.3	1.1 ± 0.4
Non-responders (within 6 weeks)	0	1	5	16

## Discussion

Several studies have demonstrated IVIG to be effective in the treatment of MG^24–26, 28^. We wanted to utilize the potential “protective” effect of IVIG to facilitate the starting of prednisone at full therapeutic doses in patients for whom pyridostigmine provided inadequate control of generalized MG. Was also wanted to determine if there was a lower rate of paradoxical exacerbations with our approach compared with the rates reported in the literature, having found that only 6.7% of our patients developed this exacerbation.

There is great disparity in the reported frequency of prednisone-induced exacerbations, with rates ranging from 21 to 75% depending on the population studied^6, 9, 18–20^. This discrepancy is partly because there is no standard definition of exacerbation and because each study uses different definitions and different treatment regimens. No previous study has used IVIG as standard prior to starting corticosteroids, but several have focused on identifying the predictors of prednisone-induced exacerbation with differing conclusions. Brunner et al. suggested that prednisone-induced exacerbation was more severe when the pre-existing MG symptoms were more severe^6^, and Chung et al. suggested that more severe exacerbations occurred in patients with infiltrating thymoma^7^. In contrast to these, however, Seybold et al. have maintained that no clinical or epidemiological predictor is sufficiently significant^18^. Indeed, some authors have suggested that prednisone-induced exacerbations are merely fluctuations of the underlying MG^10, 12^. In the article by Bae et al. the authors identified independent predictors of prednisone-induced exacerbation, such as advanced age, predominantly bulbar symptoms, and a worse baseline neurological status^19^.

Our study population was recruited by consecutive sampling, and as such, was heterogeneous in terms of demographic and MG characteristics (type and severity). To compare the incidence of exacerbations in our study population with that of the work by Bae et al., we used the same methodology with the inclusion of additional evaluation scales to allow for further analysis.

We found that combination treatment with IVIG (2 g/kg/day for 5 days) plus prednisone at a maximum dose from the first day was safe, with only two patients (4.4%) experiencing significant adverse effects from IVIG and neither requiring hospitalization. Importantly, combined IVIG and prednisone treatment was also safe for elderly patients given that 62.2% of our sample were aged > 64 years. We consider this to be key given the increase in the number of cases of MG in older people seen in recent years^45^. Furthermore, prednisone could be started at maximum daily doses from the first day of treatment rather than needing to use the gradually escalating dose recommended by some authors^18^. In turn, this allowed us to achieve a lower total dose to achieve the desired effect. Older patients and those with comorbidities (e.g., osteoporosis or diabetes mellitus) prescribed lower prednisone doses than indicated in the clinical guideline (i.e., 0.5–0.75 mg/kg/day) also achieved clinically significant improvements. Notably, the mean prednisone dose in our study was lower than in the study by Bae et al., yet we still had more treatment responders.

We used a battery of scales validated for use in MG^38–44^. Both the objective scales for physical examination (QMC and MG-composite scale) and the patient-reported scales on treatment response (activities of daily living; ADLs) have shown sufficient sensitivity to detect improvement and exacerbation. In contrast, the MSS, used by Bae et al. to predict corticosteroid-induced exacerbations, was shown to be less sensitive for detecting improvements or exacerbations in our study, where it only detected one of the three exacerbations that occurred. In our sample, only 2.2% of patients developed a paradoxical exacerbation according to the criteria used by Bae et al., although this percentage was significantly lower (p < 0.00001). However, using the more sensitive QMG or MG-Composite scales^38, 43^, this increased to 6.7% of the patients (still significantly lower than that reported by Bae et al.). It should also be noted that two patients in our study underwent thymectomy days after starting prednisone and that neither deteriorated clinically despite the added potential for deterioration of MG (surgery plus starting prednisone).

Our study has several limitations. The most important of these is that we did not have a parallel control group receiving no IVIG before starting prednisone, instead using the same methodology used by Bae et al. to allow comparisons with their historic cohort^19^. However, that cohort was demographically different from ours and was analyzed 10–20 years ago, making strict comparison difficult. Also, we included a lower proportion of patients with thymoma than Bae et al. (2 vs 22), and most importantly, our patients had a less severe previous clinical status of MG due to being treated as outpatients and required lower doses of pyridostigmine. The high percentage of patients with less severe clinical statuses and the low prevalence of thymoma in our sample may bias the study toward better outcomes. In addition, we initiated steroids after a 7–10 day wait period after IVIG therapy to ensure that we excluded patients with highly fluctuating MG, which could be mistaken for a paradoxical exacerbation. Nevertheless, excluding these could have biased our sample toward patients with more stable MG and reduced the likelihood of an exacerbation. Another limitation is the small sample size, which should have been larger to obtain sufficient statistical power. The results obtained in this study could therefore be an underestimation. There are also two probable systematic assessment biases. The first is that all examinations were performed by the same unblinded examiner who may have been biased to wanting to see clinical improvement. To minimize this bias, we used standardized scales that have good interobserver and intraobserver correlation. The second potential bias is due to the euphoric effect that can occur with prednisone in some patients; this could affect the subjective items based on patient perception in the ADL and MG-Composite scales. Despite the clear value of telephone visits, having in-person visits would have allowed us to collect data using all physical examination scales, rather than just the ADL, with some previous efficacy studies using the QMG 14–15 days after starting IVIG^23, 25, 26^. Finally, we must acknowledge that the treatment with both IVIG and prednisone is more expensive and requires the use of more clinical resources, and that many hospitals may lack the necessary resources.

If we take into account the lower prevalence of paradoxical exacerbations in our study compared with that reported by Bae et al., we can propose a hypothesis for future studies: in a patient with generalized MG, pre-treatment with IVIG before starting de novo prednisone could protect against the known risk of corticosteroid-induced paradoxical exacerbation. The present study increases the current evidence from class 5 to class 4 for the use of IVIG prior to starting corticosteroids and serves as a pilot study for future post-approval, prospective, double-blind, randomized, case–control studies to test this association scientifically.

## Conclusion

In conclusion, combined therapy with IVIG and prednisone in patients with generalized MG is safe and effective. The rate of prednisone-induced paradoxical exacerbation in our population was lower than that reported in previous literature, suggesting that IVIG could have a protective effect against such exacerbations. Further prospective research in a larger cohort is needed to confirm this protective effect.

## Supplementary information

Supplementary Information.
