# Supplementary material for: Intravenous immunoglobulins may prevent prednisone-exacerbation in myasthenia gravis
Source: Sci Rep. 2020 Aug 11;10:13497. doi: 10.1038/s41598-020-70539-4 (PMC7421901; doi:10.1038/s41598-020-70539-4)
Supplement: Supplementary file 1 — Supplementary Information. [file 41598_2020_70539_MOESM1_ESM.pdf]

Supplementary Appendix

This appendix has been provided by the authors to give readers additional information about their work.

Title: Intravenous immunoglobulins may prevent prednisone-exacerbation in myasthenia gravis.

All authors names, degrees and affiliations

Laura Diez Porras MD [1], Christian Homedes Pedret MD [1], Maria A Alberti MD[1], Valentina Vélez Santamaría MD [1,2], Carlos Casasnovas PhD, MD[1,2,3*]

1. Neuromuscular Unit. Department of Neurology. Bellvitge University Hospital, Bellvitge Biomedical Research Institute (IDIBELL), Feixa Llarga street n/n, 08907 L'Hospitalet del Llobregat (Barcelona), Spain
2. Neurometabolic Diseases Group Bellvitge Biomedical Research Institute (IDIBELL). 199 Granvia de l'Hospitalet, 08908 L'Hospitalet de Llobregat, Barcelona
3. Center for Biomedical Research on Rare Diseases (CIBERER), ISCIII, 3-5 Monforte de Lemos. Pabellón 121. 28029 Madrid Spain
4. Department of neurology. Bellvitge University Hospital. Feixa Llarga street n/n, 08907 L'Hospitalet del Llobregat (Barcelona), Spain

*Correspondence to carloscasasnovas@bellvitgehospital.cat

Index supplementary appendix

Inclusion and exclusion criteria …………..…...……………………………… page 3

Study scales ……………………………………………………………….. pages 4-8

Bibliography ……………………………………………………………….…… page 9

Supplementary Table S1. Inclusion and exclusion criteria

Inclusion criteria	Exclusion criteria
Patients older than 18 years	Ocular MG
Generalized MG according to clinical and electrophysiological criteria [63, 64] and/or antibody-positive	Pregnancy
MG from IIa to V of MGFA clinical classification system	Breastfeeding women
Patients that required first-time corticosteroid therapy for control of MG symptoms	Hypersensitivity to IVIg
	Serum IgA deficiency (<5% below lower limit of reference values)
	History of previous severe adverse event to IVIg
	Presence of diseases that could contraindicate or interfere with the treatment of oral prednisone or IVIG such as heart failure, cardiomyopathy, severe coronary heart disease, deep venous thrombosis, stages IV or V of chronic kidney disease, severe hypertension, known hypercoagulable state, history of thrombotic episodes (pulmonary embolism, acute myocardial infarction, ischaemic stroke, peripheral embolism)
	Patients who previously received immunosuppressive treatment for MG
	Patients who had received IVIg the previous 3 months
	Patients who had large worsening in their MG between the IgIV infusion and prednisone initiation (highly fluctuating MG patients)

Supplementary table S2. Myasthenia gravis severity scale (MSS) [1–3]

Myasthenia Severity Scale (MSS)	
Dyspnea	1 = Intubated<br>2 = Dyspnea at rest<br>3 = Dyspnea on exertion<br>4 = None
Cough	1 = Intubated<br>2 = Weak<br>3 = Normal
Ocular	1 = Weakness at rest<br>2 = Weakness on fatigue<br>3 = None
Bulbar	1= Weakness at rest<br>2 = Weakness on fatigue<br>3 = None
Extremities	1 = Worst affected muscle 3/5 or less<br>2 = Worst affected muscle 4/5 motor strenght or weakness on fatigue<br>3 = No detectable weakness

Table S3. Quantitative myasthenia gravis score (QMG) [4,5].

Test ítems weakness	None	Mild	Moderate	Severe	Score
**Grade**	**0**	**1**	**2**	**3**	
**Doble vision (lateral gaze) sec**	60	11-59	1-10	Spontaneous	
**Ptosis (upward gaze) sec**	60	11-59	1-10	Spontaneous	
**Facial muscles**	Normal lid closure	Complete, weak, some resistance	Complete, without resistance	Incomplete	
**Swallowing 4 oz. Water (1/2 cup)**	Normal	Minimal coughing or throat clearing	Severe coughing, choking or nasal regurgitation	Cannot swallow (test not attempted)	
**Speech following counting aloud 1-50 (onset of dysarthria)**	None at #50	Dysarthria at #30-49	Dysarthria at #10-29	Dysarthria at #9	
**Rigth arm outsrethced (90º, sitting) sec**	240	90-239	10-89	0-9	
**Left arm outsrethced (90º, sitting) sec**	240	90-239	10-89	0-9	
**Foced vital capacity**	≥ 80%	65-79%	50-64%	< 50%	
**Right hand grip (kg)**<br>**Male:**<br>**Female:**	 ≥ 45<br>≥ 30	 15-44<br>10-29	 5-14<br>5-9	 0-4<br>0-4	
**Left hand grip (kg)**<br>**Male:**<br>**Female:**	 ≥ 35<br>≥ 25	 15-34<br>10-24	 5-14<br>5-9	 0-4<br>0-4	
**Head, lifted (45º supine) Sec**	120	30-119	1-29	0	
**Right leg outstretched (45-50º, supine) Sec**	100	31-99	1-30	0	
**Left leg outstretched (45-50º, supine) Sec**	100	31-99	1-30	0	

Sec: seconds

Table S4. Myasthenia gravis activities of daily living profile (ADL) [6,7].

Items	Grade 0	Grade 1	Grade 2	Grade 3	Score
**1. Talking**	Normal	Intermitent slurring or nasal speech	Constant slurring or nasal speech, but can be understood	Difficult to understand speech	
**2. Chewing**	Normal	Fatigue with solid food	Fatigue with soft food	Gastric tube	
**3. Swallowing**	Normal	Rare episode of choking	Frequent choking, necessitating changes in diet	Gastric tube	
**4. Breathing**	Normal	Shortness of breath with extertion	Shortness of breath at rest	Ventilator dependence	
**5. Impairment of ability to brush teeth or comb hair**	None	Extra effort, but no rest periods needed	Rest periods needed	Cannot do one of theses functions	
**6. Impairment of ability to arise from a chair**	None	Mild, sometimes uses arms	Moderate, always uses arms	Severe, requires assistance	
**7. Double vision**	None	Occurs, but not daily	Daily, but not constant	Constant	
**8. Eyelid droop**	None	Occurs, but not daily	Daily, but not constant	Constant	

Table S5. The myasthenia-gravis composite scale (MG-Composite).[8,9]

**Ptosis, upward gaze** (physician examination)	>45 seconds = 0	11-45 seconds = 1	1-10 seconds = 2	Immediate = 3	
**Double vision on lateral gaze, right or left** (physician examination)	>45 seconds = 0	11-45 seconds = 1	1-10 seconds = 3	Immediate = 4	
**Eye closure** (physician examination)	Normal = 0	Mild weakness (can be forced open with effort) = 0	Moderate weakness (can be forced open easily) =1	Severe weakness (unable to keep eyes closed) = 2	
**Talking** (patient history)	Normal = 0	Intermitent slurring or nasal speech = 2	Constant slurring or nasal but can be understood = 4	Difficult to understant speech = 6	
**Chewing** (patient history)	Normal = 0	Fatigue with solid food = 2	Fatigue with soft food =4	Gastric tube = 6	
Swallowing (patient history)	Normal = 0	Rare episode of choking or trouble swallowing = 2	Frequent trouble swallowing (e.g. necessitating changes in diet) = 5	Gastric tube = 6	
**Breathing** (thought to be caused by MG)	Normal = 0	Shortness of breath with exertion = 2	Shortness of breath at rest = 4	Ventilator dependence = 9	
**Neck flexion or extension (weakest)** (physician examination)	Normal = 0	Mild weakness = 1	Moderate weakness (i.e. - 50% weak. +/- 15%) = 3	Severe weakness = 4	
**Shoulder abduction** (physician examination)	Normal = 0	Mild weakness = 2	Moderate weakness (i.e. - 50% weak. +/- 15%) = 4	Severe weakness = 5	
**Hip flexion** (physician examination)	Normal = 0	Mild weakness = 2	Moderate weakness (i.e. - 50% weak. +/- 15%) = 4	Severe weakness = 5	
				**Total score**	

Please note that “moderate” weakness for for neck and limb ítems should be construed as weakness that equals roughly 50% +/- 15% of expected normal strength. Any weakness milder than that would be “mild” and any weakness more severe tan that would be classified as “severe”.

Table S6. 15-item myasthenia gravis quality of life scale (MG-QOL15) [10].

Please indicate how true each statement has been (over the past 4 weeks).

	**Not at all**	**A little bit**	**Some-what**	**Quite a bit**	**Very much**	
	0	1	2	3	4	
1.I am frustrated by my condition						
2. I have trouble using my eyes						
3. I have trouble eating						
4. I have limited my social activity because of my condition						
5. My condition limits my ability to enjoy hobbies and fun activities						
6. I have trouble meeting the needs of my famililiy						
7. I have to make plans around my condition						
8. My occupational skills and job status have been negatively affected						
9. I have difficulty speaking						
10. I have trouble driving						
11. I am depressed about my condition						
12. I have trouble walking						
13. I have trouble getting around public places						
14. I feel overwhelmed by my condition						
15. I have trouble performing my personal grooming needs						
**Total score**						

Bibliography

1. Bae, J. S., Go, S. M. & Kim, B. J. Clinical predictors of steroid-induced exacerbation in myasthenia gravis. *J. Clin. Neurosci.* **13**, 1006–1010 (2006).
2. Qureshi, A. I. *et al.* Plasma exchange versus intravenous immunoglobulin treatment in myasthenic crisis. *Neurology* **52**, 629–632 (1999).
3. Rosenfeld J, Jackson CE. Quantitative assessment and outcome measures in neuromuscular disease. In: Katirji B, Kaminski HJ, Preston DC, et al., editors. Neuromuscular Disorders in Clinical Practice. 1st ed. Woburn: Butterworth-Heinemann, p. 309–43 (2002).
4. Barohn, R. J. *et al.* Reliability Testing of the Quantitative Myasthenia Gravis Scorea. *Ann. N. Y. Acad. Sci.* **841**, 769–772 (1998).
5. Katzberg, H. D., Barnett, C., Merkies, I. S. J. & Bril, V. Minimal clinically important difference in myasthenia gravis: Outcomes from a randomized trial. *Muscle and Nerve* **49**, 661–665 (2014).
6. Wolfe, G. I. *et al.* Myasthenia gravis activities of daily living profile. *Neurology* **52**, 1487–1489 (1999).
7. Muppidi, S. The Myasthenia Gravis-Specific Activities of Daily Living Profile. *Ann. N. Y. Acad. Sci.* **1274**, 114–119 (2012).
8. Burns, T. M., Conaway, M. & Sanders, D. B. The MG Composite: A valid and reliable outcome measure for myasthenia gravis. *Neurology* **74**, 1434–1440 (2010).
9. Burns, T. M. The MG composite: An outcome measure for myasthenia gravis for use in clinical trials and everyday practice. *Ann. N. Y. Acad. Sci.* **1274**, 99–106 (2012).
10. Burns, T. M., Grouse, C. K., Wolfe, G. I., Conaway, M. R. & Sanders, D. B. The MG-QOL15 for following the health-related quality of life of patients with myasthenia gravis. *Muscle and Nerve* **43**, 14–18 (2011).
